# Comparison of the target-controlled infusion and the manual infusion of propofol anesthesia during electroconvulsive therapy: an open-label randomized controlled trial

**DOI:** 10.1186/s12888-021-03069-6

**Published:** 2021-02-04

**Authors:** Meng-Ling Hsieh, Yen-Ting Lu, Chih-Chung Lin, Chin-Pang Lee

**Affiliations:** 1 Department of Anesthesiology, Chang Gung Memorial Hospital, Linkou, Taoyuan, Taiwan; 2 School of Medicine, Chang Gung University, Taoyuan, Taiwan; 3 Department of Psychiatry, Chang Gung Memorial Hospital, Linkou, Taoyuan, Taiwan

**Keywords:** Electroconvulsive therapy (ECT), Target-controlled infusion (TCI), Manual infusion (MI), Propofol

## Abstract

**Background:**

Target-controlled infusion (TCI) of propofol is a well-established method of procedural sedation and has been used in Japan for anesthesia during electroconvulsive therapy (ECT). However, the usefulness of the TCI of propofol for ECT has yet to be determined. This study aimed to compare the TCI and manual infusion (MI) of propofol anesthesia during ECT.

**Methods:**

A total of forty psychiatric inpatients receiving bitemporal ECT were enrolled in the present study and randomized into the TCI group (N = 20) and the MI group (N = 20). Clinical Global Impression (CGI) and Montreal Cognitive Assessment (MoCA) scores were measured before and after ECT. The clinical outcomes, anesthesia-related variables, and ECT-related variables were compared between the two groups. Generalized estimating equations (GEEs) were used to model the comparison throughout the course of ECT.

**Results:**

A total of 36 subjects completed the present study, with 18 subjects in each group. Both the groups didn’t significantly differ in the post-ECT changes in CGI and MoCA scores. However, concerning MoCA scores after 6 treatments of ECT, the MI group had improvement while the TCI group had deterioration. Compared with the MI group, the TCI group had higher doses of propofol, and longer procedural and recovery time. The TCI group seemed to have more robust seizures in the early course of ECT but less robust seizures in the later course of ECT compared with the MI group.

**Conclusions:**

The present study does not support the use of TCI of propofol for anesthesia of ECT.

**Trial registration:**

(ClinicalTrials.gov): NCT03863925. Registered March 5, 2019 - Retrospectively registered.

**Supplementary Information:**

The online version contains supplementary material available at 10.1186/s12888-021-03069-6.

## Background

Propofol has been widely used as an anesthetic agent during electroconvulsive therapy (ECT) [1, 2]. Compared with the use of barbiturates and ketamine as anesthetics during ECT, the use of propofol not only results in comparable clinical outcomes but also has distinct advantages in terms of favorable hemodynamics and improved post-ECT recovery, [1, 3] although propofol is associated with a shorter duration of seizure [2, 4].

Target-controlled infusion (TCI) of propofol has been widely used in clinical practice [5–10]. Effect-site concentrations of propofol are calculated a priori according to pharmacokinetic algorithms, which are derived and validated from population samples [8]. The rate of the infusion of propofol is automatically controlled [11]. Compared with the manual infusion (MI) of propofol, the TCI of propofol is associated with a more stable hemodynamic profile during anesthesia [5] and a shorter recovery time after anesthesia, [6, 7] although the TCI of propofol may be associated with a higher total dose of propofol [9, 12].

MI is universally adopted for administration of anesthetic agents in ECT [3]. The primary considerations of anesthesia for ECT include safety, tolerability, cost, mitigation of adverse hemodynamic changes, facilitation of therapeutic seizures, improvement of emergence of anesthesia, and protection of cognitive adverse effects. As the TCI of propofol may provide better hemodynamic management and more speedy post-anesthesia recovery [5–7], it is of interest to adopt the TCI of propofol in anesthesia for ECT. In addition, the use of a TCI device is not only able to provide instantaneous information of the effect-site concentrations of propofol, but also to maintain steady effect-site concentrations of propofol at time of electric stimulus. Recently, three studies in Japan have explored the use of the TCI of propofol for anesthesia of ECT [13–15]. The TCI of propofol may be useful for patients receiving ECT when succinylcholine is contraindicated [13, 14]. In addition, with use of a TCI system, Imashuku et al. showed that a higher effect-site concentration of propofol was associated with better early memory improvement after ECT [15]. However, propofol may increase seizure threshold, and accordingly a higher effect-site concentration of propofol could impede elicitation of therapeutic seizures and lower efficacy of ECT. In addition, the TCI of propofol may deliver a higher total dose of propofol than the MI of propofol [9, 12]. It would take a longer time to emergence with a higher total dose of propofol. The use of a TCI device may prolong the duration of ECT at least two points, setting up the device and waiting for emergence. It would increase burden of nursing care and impede workflow. Moreover, the use of a TCI device may make ECT more costly than the use of MI. Therefore, although the TCI of propofol could be useful in anesthesia of ECT, it warrants further research to clarify the role of the TCI of propofol in anesthesia for ECT.

To the best of our knowledge, there is no study that directly compares the TCI of propofol with the MI of propofol in anesthesia of ECT. In the present study, we hypothesized that the TCI of propofol may be non-inferior to the MI of propofol in anesthesia of ECT in terms of the effects on clinical variables such as therapeutic efficacy, seizure threshold, cognitive side effects, hemodynamic parameters, and the speed of recovery after ECT.

## Methods

The present study was approved by the institutional review board (IRB) of the Chang Gung Medical Foundation (IRB: 201700862A3). Participants were recruited from the acute psychiatric ward at Chang Gung Memorial Hospital, Linkou, between August 2017 and September 2018. All participants and their legal guardians gave written informed consent to ECT and participation in the present study. The inclusion criteria were as follows: (1) age from 20 to 65 years; (2) a clinical diagnosis of major depressive disorder, bipolar disorder or schizophrenia; and (3) normal or corrected vision and hearing. The exclusion criteria were as follows: (1) past or current diagnosis of a neurocognitive disorder; (2) any contraindication to ECT within one month before ECT, such as myocardial infarction, cerebrovascular disease, increased intracranial pressure, pheochromocytoma, and unstable vertebral fracture; (3) untreated substance use order; and (4) lack of cooperation. Regarding medications, lithium carbonate and anticonvulsants were discontinued before ECT. Antidepressant and antipsychotic medications were maintained at the same doses throughout ECT treatment. For those who were receiving benzodiazepine drugs before ECT, benzodiazepine drugs were discontinued or tapered to a lower dose if tolerated. For individual subjects, the total number of ECT treatments was decided by his/her attending psychiatrist.

### Sample and study design

**Fig. 1:**
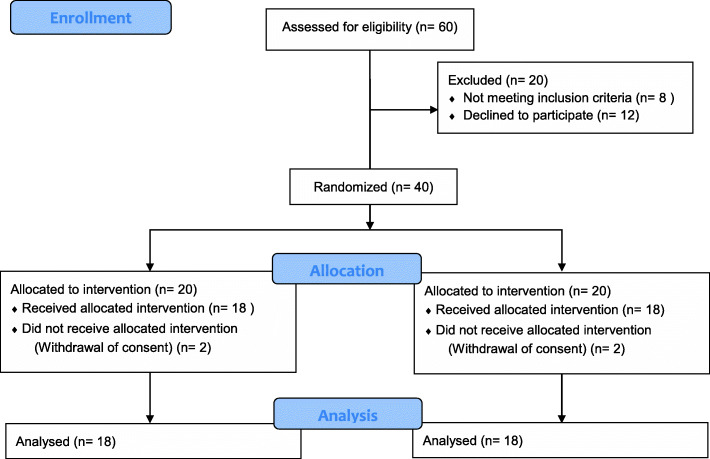
Flow diagram of the study participants

The present study adopted a permutation block randomization design with two groups: the TCI group and the MI group. One of the authors, MLH, generated the random allocation sequence. One of the authors, CPL, enrolled participants. Given a power of 80% and a level of significance of 5%, we assumed that the mean difference in the total propofol amount between both groups would be 20 mg and the pooled standard deviation would be 20 mg. Hence, the sample size of each group should be 16. Considering dropouts, we decided the sample size of each group should be 20. The treatment team, consisting of the attending psychiatrist and anesthesiologist, was not blinded to patient allocation. A total of forty subjects were enrolled in the present study. With randomization, 20 subjects were allocated to The TCI group, and 20 were allocated to The MI group. Four subjects dropped out of the present study due to their withdrawal of their informed consent. A total of 36 subjects completed the present study, and both groups were composed of 18 subjects (Fig. 1).

### Instruments

#### Clinical global impression (CGI)

The CGI is a well-validated scale that is widely used in psychiatric research [16]. It consists of CGI-severity (CGI-S) and CGI-improvement (CGI-I). The CGI-S assesses the severity of illness and is a 7-point Likert-type scale in which “1” stands for “Normal, not at all ill”, “2” for “Borderline mentally ill”, “3” for “Mildly ill”, “4” for “Moderately ill”, “5” for “Markedly ill”, “6” for “Severely ill”, and “7” for “Among the most extremely ill patients”. The CGI-I assesses the total improvement due to treatment and is a 7-point Likert-type scale in which “1” stands for “Very much improved”, “2” for “Much improved”, “3” for “Minimally improved”, “4” for “No change”, “5” for “Minimally worse”, “6” for “Much worse”, and “7” for “Very much worse”. For each patient, the reference point of assessment of CGI-I scores was his/her severity of illness on admission.

#### Montreal cognitive assessment (MoCA)

The MoCA is a reliable and well-validated measure of global cognitive ability [17]. The MoCA is useful for monitoring cognitive impairment in patients receiving ECT [18]. The MoCA is a 30-point test and assesses multiple domains of cognition, such as short-term memory and visuospatial abilities. In the present study, we used the Taiwanese version of the MoCA [19].

#### The Observer’s assessment of alertness/sedation scale (OAA/S)

The OAA/S is a reliable and valid measure of the level of alertness in subjects who are sedated [20]. The OAA/S is a 5-point Likert-type scale in which “5” stands for “Responds readily to name spoken in normal tone”, “4” for “Lethargic response to name spoken in normal tone”, “3” for “Response only after name is called loudly and/or repeatedly”, “2” for “Response only after mild prodding or shaking”, “1” for “Response only after painful trapezius squeeze”, and “0” for “No response after painful trapezius squeeze”. A score on the OAA/S less than or equal to 3 is considered sufficient for anesthesia sedation.

#### Aldrete score

The Aldrete score is a scale commonly used to clinically assess the physical status of patients recovering from anesthesia and to follow their awakening process [21]. The Aldrete score has 5 items (activity, respiration, circulation, consciousness and oxygen saturation), with each item rated as 0, 1 or 2, giving a maximum possible score of 10. A patient with an Aldrete score of 9 or 10 is considered suitable to be discharged from the postanesthesia recovery room.

### Target-controlled and manual infusion of propofol

In the TCI group, an Injectomat TIVA Agilia device (Fresenius Kabi, Cheshire, UK) was used for the infusion of propofol with the Schnider model [22]. The parameters for the determination of the infusion rate included age, sex, and body weight. The desired effect-site concentration of propofol (C_e_) was defined as the minimal effect-site concentration of propofol necessary to produce an adequate depth of sedation, as evidenced by an OAA/S score less than or equal to 3. In the first ECT treatment, the initial target effect-site concentration of propofol was set at 1.5 μg/mL and titrated up in increments of 0.1 μg/mL until the desired C_e_ was achieved. During consecutive ECT treatments, the target effect-site concentration of propofol was set at the previous desired C_e_ and titrated up in increments of 0.1 μg/mL as appropriate. Titration procedures were identical for all subjects in the TCI group. The TCI of propofol was stopped just before the administration of the electric stimulus. In the MI group, propofol was manually infused with an initial bolus of 1 mg/kg, and titrated up with each step of increments of 10 mg until the desired level of sedation, an OAA/S score less than or equal to 3, was achieved. For between-group comparisons, the predicted blood levels of propofol in The MI group were calculated using iTIVA Anesthesia Plus [23]. The total dose of propofol was the total amount of propofol divided by the ideal body weight of a subject. The unit of the total dose of propofol was expressed in mg per kilogram (mg/kg).

### ECT and anesthesia procedures

Bitemporal brief-pulse (1 ms) ECT was performed 3 days per week using a MECTA Spectrum 5000Q device (Mecta Corp., Lake Oswego, OR; maximum output, 576 mC). Anesthesia was induced with propofol and succinylcholine (1–2 mg/kg). For each subject, the type of infusion of propofol was determined by randomization as aforementioned. Anesthesiologists administered anesthesia as per their usual clinical practice: (1) initial preoxygenation at 100% oxygen, (2) intravenous induction with propofol and succinylcholine, (3) hyperventilation using 100% oxygen by bag-valve-mask before the administration of the electric stimulus, and (4) bag-valve-mask ventilation using 100% oxygen until the subject resumed spontaneous breathing and the sedation level returned to an OAA/S score greater than or equal to 4. The anesthetic-ECT time interval (ASTI) was measured from the commencement of the propofol infusion to the start of the ECT stimulus for each ECT session using a stopwatch [24].

The seizure threshold was measured at the first ECT session and was defined as the minimum electrical intensity needed to produce a generalized seizure of any duration, evidenced as a motor seizure (using the isolated cuffed limb technique on the right leg) and/or ictal EEG manifestation (slow wave activity) [25]. The titration procedure was identical for all subjects, starting at the minimum energy level (24 mC). Up to 4 stimulations were permitted in a session. Two frontomastoid electroencephalographic (EEG) channels were used for EEG recording. After the seizure threshold was determined, the stimulus dose at consecutive ECT treatments was given at 1.5 times the seizure threshold. A further increase in stimulus dose was permitted if there was poor seizure quality or if there was an inadequate clinical response.

### Clinical evaluation

The psychiatrist (CPL) rated the CGI and MoCA for each subject before the first ECT session, weekly during the ECT course, and after the end of the ECT course. In addition, the following ECT- and treatment-related variables were recorded for each ECT treatment: total amount of propofol (mg), total amount of succinylcholine (mg), stimulus dose (mC), initial seizure threshold (mC), motor and EEG seizure durations, seizure adequacy (range: 0–100%), [26] ECT treatment number (order of the treatment in the ECT course), and American Society of Anesthesiologists (ASA) class. Heart rates (HRs), systolic blood pressures (SBPs), and diastolic blood pressures (DBPs) were recorded at three time points, namely, baseline, during ictal phase, and before patient transferal to the postanesthesia recovery room (discharge). The recovery time was measured from the cessation of anesthesia to the achievement of adequate recovery, defined by an Aldrete score of 9 or 10. The recovery time was measured using a stopwatch.

### Statistical analysis

The primary outcome measure was the total dose of propofol which provided sufficient depth of anesthesia for ECT. The secondary outcomes were clinical variables such as therapeutic efficacy, seizure threshold, cognitive side effects, hemodynamic parameters, and the speed of recovery after ECT. Response was defined as a CGI-S score less than or equal to 3. Statistical analysis was performed using IBM Statistical Package for the Social Sciences (SPSS) version 22 for Windows (IBM, Somers, NY USA). Chi-square tests and *t* tests were used as appropriate. Generalized estimating equations (GEEs) were modeled to determine the correlations among the parameters within the subjects [27–29]. GEEs were fit with the first-order autoregressive (AR1) or exchangeable model to find the best structure. A total of thirteen models of GEEs were modelled, and the dependent variables were total dose of propofol, stimulus dose, recovery time, ASTI, seizure adequacy, EEG seizure duration, motor seizure duration, the difference between ictal HR and baseline HR (ictal HR – baseline HR), the difference between discharge HR and baseline HR (discharge HR – baseline HR), the difference between ictal SBP and baseline SBP (ictal SBP – baseline SBP), the difference between discharge SBP and baseline SBP (discharge SBP – baseline SBP), the difference between ictal DBP and baseline DBP (ictal DBP – baseline DBP), and the difference between discharge DBP and baseline DBP (discharge DBP – baseline DBP), respectively. Predictor variables included group (TCI versus MI), sex (male versus female), age, ECT treatment number, and interaction between group and ECT treatment number. Probability distribution was normal distribution. A *P* value less than 0.05 was considered significant.

## Results

**Fig. 2:**
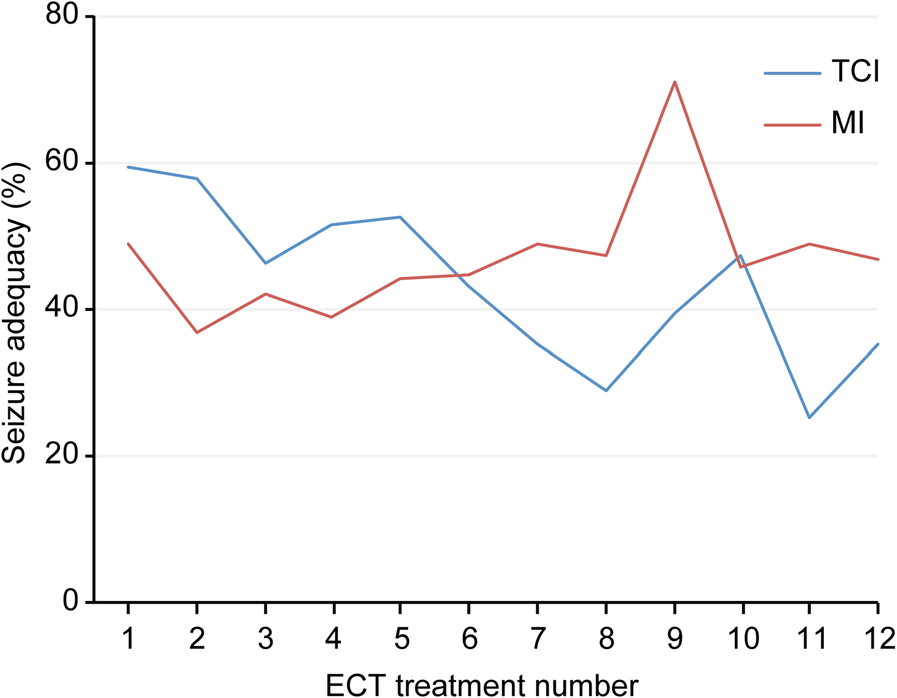
Seizure adequacy and ECT treatment number in the two groups. The TCI group had greater seizure adequacy before the sixth ECT treatment and lower seizure adequacy afterwards compared with the MI group

The demographic and clinical characteristics were comparable between the two groups except for the anesthesia-related factors (Table 1). Compared with the MI group, the TCI group had significantly higher doses of propofol, higher predicted effect-site levels of propofol, lower doses of succinylcholine, a longer ASTI, and a longer recovery time. Both the group did not significantly differ in seizure threshold, the total number of ECT treatments, stimulus dose, and seizure adequacy. Table 2 shows the clinical outcomes in both the groups before and after the course of ECT. Both groups had significant improvements in CGI scores after ECT. The MI group had trend towards improvement in MoCA scores after the course of ECT (*p* = 0.052). There was no difference between the two groups in CGI and MoCA scores after ECT (Table 2). However, the TCI group had numerically worse MoCA scores after 9 treatments of ECT while the MI group had numerically better MoCA scores after 6 treatments of ECT (Table 3). Table 4 shows the abridge summary of GEE models. Compared with the MI group, the TCI group had significantly higher total dose of propofol, longer recovery time and ASTIs, better seizure adequacy, and longer EEG seizure duration than the MI group. The ECT treatment number positively predicted stimulus dose, and negatively predicted the difference between discharge HR and baseline HR (discharge HR – baseline HR). Compared with the interaction between the MI group and the ECT treatment number, the interaction between the TCI group and the ECT treatment number positively predicted stimulus dose and the difference between ictal DBP and baseline DBP (ictal DBP – baseline DBP), and negatively predicted seizure adequacy. Age negatively predicted EEG seizure duration and the difference between ictal HR and baseline HR (ictal HR – baseline HR). Gender negatively predicted the differences between ictal SBP and baseline SBP, discharge SBP and baseline SBP, and ictal DBP and baseline DBP. Figure 2 shows that the TCI group had greater seizure adequacy before the sixth ECT treatment and lower seizure adequacy afterwards compared with the MI group.

**Table 1:** Sociodemographic and clinical characteristics

	Target-controlled infusion (TCI)	Manual infusion (MI)	
	(*N* = 20)	(N = 20)	*P*
Male/Female	6/12	12/6	0.11
Age, yr, mean ± SD	38.1 ± 13.8	40.0 ± 11.8	0.60
Weight, kg, mean ± SD	63.5 ± 16.7	70.9 ± 15.1	0.15
Height, cm, mean ± SD	160.8 ± 8.0	164.7 ± 9.8	0.18
BMI, kg/m^2^, mean ± SD	24.4 ± 5.5	26.3 ± 5.4	0.29
ECT factors			
Total number of ECT treatments, mean ± SD	7.2 ± 3.2	7.6 ± 2.8	0.68
Seizure threshold, mC, mean ± SD	100.0 ± 31.1	96.8 ± 50.5	0.81
Stimulus dose of the last treatment, mC, mean ± SD	306.7 ± 176.2	251.6 ± 136.1	0.30
Seizure adequacy, %, mean ± SD	48.0 ± 28.7	45.2 ± 28.6	0.40
Anesthesia-related factors			
ASA class 1–2/ASA class 3	16/4	18/2	0.66
Predicted blood level of propofol, μg/mL, mean ± SD	3.16 ± 0.86	2.71 ± 0.78	< 0.001
Total dose of propofol, mg/kg, mean ± SD	1.31 ± 0.60	1.06 ± 0.22	< 0.001
Succinylcholine, mg/kg, mean ± SD	1.10 ± 0.30	1.20 ± 0.28	0.002
ASTI, min, mean ± SD	4.55 ± 1.68	3.63 ± 1.29	< 0.001
Recovery time, min, mean ± SD	7.98 ± 8.83	5.84 ± 4.76	0.015
Diagnosis, N (%)			
Schizophrenia	8 (40%)	8 (40%)	1
Bipolar disorder	5 (25%)	7 (35%)	0.49
Major depressive disorder	7 (35%)	5 (25%)	0.49
Psychotropic medication, N (%)			
Antipsychotics	17 (85%)	15 (75%)	0.70
Antidepressants	9 (45%)	8 (40%)	0.75
Benzodiazepines	8 (40%)	8 (40%)	1
Clinical scales, baseline, mean ± SD			
CGI-S	5.39 ± 0.92	5.22 ± 0.73	0.55
CGI-I	4.78 ± 0.88	4.78 ± 0.94	1
MoCA	18.2 ± 9.7	20.2 ± 8.6	0.52

**Table 2:** Comparisons between the TCI group (N = 18) and the MI group (*N* = 18)

					Within-group				Between-group	
	Baseline		Post-ECT		TCI		MI		TCI vs MI	
	TCI	MI	TCI	MI	*P*	Cohen’s d [95% CI]	*P*	Cohen’s d [95% CI]	*P*	Cohen’s d [95% CI]
MoCA, mean ± SD	18.2 ± 9.7	20.3 ± 8.6	20.3 ± 8.2	24.3 ± 4.0	0.139	0.23 [− 0.43, 0.89]	0.052	0.60 [− 0.09, 1.27]	0.48	− 0.24 [− 0.89, 0.42]
CGI-S, mean ± SD	5.39 ± 0.92	5.22 ± 0.73	2.72 ± 1.49	2.22 ± 0.73	< 0.001	− 2.16 [− 3.13, −1.17]	< 0.001	−4.11 [−5.61, − 2.58]	0.44	0.26 [− 0.41, 0.91]
CGI-I, mean ± SD	4.78 ± 0.88	4.78 ± 0.94	1.89 ± 0.68	1.67 ± 0.59	< 0.001	− 3.68 [− 5.05, − 2.28]	< 0.001	−3.96 [− 5.42, − 2.48]	0.56	0.19 [− 0.46, 0.85]
CGI-S ≤ 3, N (%)	0 (0)	0 (0)	9 (50)	13 (72)					0.30	− 0.53 [− 1.29, 0.24]
CGI-I < 3, N (%)	0 (0)	0 (0)	15 (83)	17 (94)					0.60	−0.67 [−1.98, 0.63]

*TCI* target-controlled infusion, *MI* manual infusion

**Table 3:** The comparisons of MoCA scores throughout ECT treatments

	TCI group (mean ± SD)	*N*	*p*-value^a^	MI group (mean ± SD)	*N*	*p*-value^a^
Baseline	18.2 ± 9.7	18	–	20.2 ± 8.6	18	–
After 3 treatments	20.6 ± 8.0	18	0.061	20.8 ± 8.6	18	0.210
After 6 treatments	20.9 ± 7.5	18	0.074	23.6 ± 6.4	18	0.035
After 9 treatments	15.9 ± 10.0	6	0.571	22.9 ± 8.1	9	0.086
After 12 treatments	12.8 ± 11.2	4	1	23.7 ± 5.5	3	0.264

^a^: paired sample t test

**Table 4:** Tests of Model Effects between groups and ECT treatment times using generalized estimating equations (GEEs)

	TCI versus MI	ECT treatment number	TCI × ECT treatment number versus MI × ECT treatment number	Age	Male versus Female
	β (SE)	β (SE)	β (SE)	β (SE)	β (SE)
Total dose of propofol (mg/kg)^a^	1.82 (0.22)***	0.02 (0.02)	0.02 (0.03)	−0.00 (0.01)	0.29 (0.16)
Stimulus dose (mC)	−2.94 (33.6)	22.53 (2.54)***	10.82 (3.64)**	0.68 (1.13)	9.82 (29.6)
Recovery time (min)	5.09 (2.17)*	−0.10 (0.26)	−0.44 (0.38)	0.06 (0.05)	2.40 (1.26)
ASTI (min)	0.74 (0.32)*	−0.01 (0.04)	0.03 (0.06)	0.00 (0.01)	0.42 (0.17)*
Seizure adequacy (%)	21.82 (7.35)**	−2.42 (0.92)*	−3.78 (1.28)**	0.01 (0.16)	6.23 (3.95)
EEG seizure duration (sec)	9.91 (5.96)*	−0.00 (0.73)	−2.03 (1.04)	−0.27 (0.13)*	− 0.62 (3.31)
Motor seizure duration (sec)	0.45 (4.75)	−0.84 (0.57)	0.23 (0.81)	−0.15 (0.11)	0.35 (2.85)
Ictal HR – baseline HR _(_beats/min)	−4.53 (8.42)	0.21 (0.96)	−0.26 (1.37)	−0.50 (0.22)*	−3.89 (5.60)
Discharge HR - baseline HR _(_beats/min)	−7.59 (5.68)	−2.06 (0.69)**	0.02 (0.98)	−0.14 (0.13)	1.39 (3.25)
Ictal SBP– baseline SBP (mmHg)	−11.28 (6.88)	1.01 (0.84)	2.01 (1.19)	−0.23 (0.15)	−17.86 (3.92)***
Discharge SBP– baseline SBP (mmHg)	−6.64 (6.87)	0.77 (0.83)	0.95 (1.18)	0.00 (0.16)	−9.40 (4.04)*
Ictal DBP– baseline DBP (mmHg)	−8.92 (5.07)	−1.00 (0.62)	2.18 (0.88)*	−0.19 (0.11)	−7.10 (2.88)*
Discharge DBP– baseline DBP (mmHg)	−2.97 (4.15)	−0.17 (0.51)	0.07 (0.73)	−0.12 (0.09)	−2.81 (2.24)

*: *p* < 0.05; **: *p* < 0.01; ***: *p* < 0.001

^a^: Total dose of propofol was total amount of propofol divided by ideal body weight

*ASTI* anesthetic-stimulation time interval, *EEG* electroencephalogram, *HR* heart rate, *SBP* systolic blood pressure, *DBP* diastolic blood pressure

## Discussion

In the present study, we demonstrated that the TCI of propofol was less preferable than the MI of propofol for patients receiving ECT in terms of efficacy, cognitive adverse effect, hemodynamic profile, and cost-effectiveness. First, TCI consumed higher total doses of propofol for induction than MI. Second, TCI was less cost-effective than MI. It took more time achieving adequate depth of anesthesia as well as recovering from anesthesia in the TCI group than in the MI group. Therefore, TCI was less time-efficient than MI. Third, the MI group had numerically higher response rates than the TCI group despite TCI was associated with higher seizure adequacy and longer EEG seizure duration than MI. It suggests that TCI might reduce efficacy of ECT. Fourth, although both the groups did not differ in post-ECT changes in MoCA scores, the MI group had a trend improvement in MoCA scores after ECT, and such improvement was statistically significant after 6 treatments of ECT. In contrast, the TCI group not only had numerically worse MoCA scores than the MI group after ECT, but also had more decline in MoCA scores in the later course of ECT. Finally, considering significant interaction between TCI and ECT treatment number, it suggests that TCI could elevate more seizure threshold than MI with each ECT treatment. The advantage of TCI in eliciting effective seizures over MI waned in the later course of ECT.

Our study raises concern that TCI of propofol might reduce efficacy of ECT. As both the groups did not differ in the total numbers of treatment, seizure thresholds, and final doses of electric stimuli, efficacy should be comparable in both the groups. Such potential inferiority in efficacy in the TCI group might be explained by higher doses of propofol as well as higher predicted blood levels of propofol in the TCI group. Propofol has anticonvulsant properties, and a higher dose of propofol may impede the elicitation of seizures by ECT [30]. As regards significant interaction between TCI and ECT treatment number in seizure adequacy, the TCI and MI groups crossed approximately at the seventh treatment of ECT. Prior to 7 treatments of ECT, the seizure adequacy was better in the TCI group, while the seizure adequacy was better in the MI group since 7 treatments (Fig. 2). The trend of declining seizure adequacy in the TCI group was not offset by increasing stimulus dose. As a higher quality seizure may be associated with a more robust clinical response [24, 26], the worse seizure adequacy could explain the inferior response rate in the TCI group. The trend of declining seizure adequacy in the TCI group throughout the course of ECT could be explained by changes in blood-brain barrier (BBB) permeability. ECT enhances BBB permeability, and the duration of BBB permeability enhancement varies from 2 to 48 h [31, 32]. As the frequency of ECT treatment was thrice weekly, the BBB permeability enhancement might consecutively decrease the sedation threshold, which is the propofol concentration required to achieve similar sedation levels. As the total dose of propofol was higher in the TCI group, the dose of propofol in the TCI group could be too much in the later course of ECT. Therefore, the TCI group could have much higher seizure threshold than the MI group in the later course of ECT.

The MI group seemed to have a trend of improvement in global cognition after bitemporal ECT, while the TCI group seemed to have a trend of decline. Previous studies showed that propofol may have no negative effect on cognitive functioning [1, 3]. A higher blood level of propofol may even be associated with early memory recovery after ECT [15]. As the TCI group had higher doses and higher predicted blood levels of propofol than the MI group, propofol-related cognitive protection would be greater in the TCI group than in the MI group. However, our findings suggest that a higher effect-site concentration of propofol causes more cognitive adverse effects. Although both the group had comparable initial seizure thresholds as well as electric stimulus doses, an excessive effect-site concentration of propofol could still impede the elicitation of therapeutic seizures by ECT, thereby decreasing the efficacy of ECT [24]. The deleterious impact of a higher effect-site concentration of propofol on seizure adequacy and MoCA score was evident after 6 treatments of ECT (Fig. 2 and Table 3). The cognitive improvement in the MI group might be explained by more robust seizures in the later course of ECT. Robust seizures might lead to more improvement in mental disorders, thereby contributing to further cognitive improvement. Another possibility is that propofol might cause cognitive dysfunction. Propofol may cause postoperative delirium by reduction of cholinesterase activity, and propofol treatment may also induce significant and persistent epigenetic changes in the brain [33]. Repeated exposure to high effect-site concentrations of propofol might induce unwanted changes in the brain. In other words, the TCI of propofol might cause more problems in post-ECT cognitive recovery than the MI of propofol by giving ‘too’ high doses of propofol.

Our study suggests that the TCI of propofol was less favorable than the MI of propofol for hemodynamic profile in ECT. With increasing ECT treatment number, both the groups had similar decrease in postictal heart rate but the TCI group had more increase in postictal diastolic blood pressure than the MI group. Our findings oppose that TCI of propofol provide better stable hemodynamic profile than MI of propofol [5]. Recently, Azuma et al. showed that increases in postictal blood pressure and heart rate may be predictive of the therapeutic efficacy of ECT for depression [34]. A robust postictal cardiac response may reflect a high-quality seizure. However, the trend of increasing postictal DBP with paralleled the trend of declining seizure adequacy in the TCI group (Table 4). Our findings suggest that increases in postictal diastolic blood pressure might indicates a poor-quality seizure. The effects of ECT treatment number, age, and sex on changes of postictal heart and blood pressures could be explained by the increase in seizure threshold commonly seen in ECT [35]. Seizure threshold would be higher in the later course of ECT, elderly, and men.

Our study suggests that TCI of propofol was less cost-effective than MI of propofol for anesthesia in ECT. The consumption of propofol was higher for TCI than MI. Although the TCI group received lower doses of succinylcholine than the MI group, but the difference seemed trivial clinically. TCI of propofol was more time-consuming than MI of propofol. First, it takes time to set up TCI in each treatment of ECT. Second, the ASTI were significantly longer in the TCI group than in the MI group. Regarding the MI of propofol, the ASTI has been advocated as a surrogate marker of the effect-site concentration of propofol; that is, a longer ASTI indicates a lower effect-site concentration of propofol [24]. It has been suggested that ASTI should be extended as long as possible to facilitate the elicitation of high-quality seizures [24], while an excessively long ASTI would lead to unwanted awakening during ECT. The use of a TCI device might help determine optimal ASTI by estimating the effect-site concentrations of an anesthetic agent. It suggests that MI of propofol is more efficient to achieve sufficient levels of anesthesia than TCI of propofol. As regards MI of propofol, the peak concentration of propofol is sufficient for induction, so anesthesiologists can administer succinylcholine to patients without delay. In contrast, in the case of TCI of propofol, the concentrations of propofol build up slowly, so anesthesiologists have to wait for sufficient sedation and then administer succinylcholine. Third, the TCI group recovered from anesthesia more slowly than the MI group. A higher dose of propofol and a higher effect-site concentration of propofol may explain the delayed recovery in the TCI group. Delayed recovery could further increase burden in post-anesthesia nursing care. In brief, TCI of propofol was more costly in propofol dose, time, and care than MI of propofol in anesthesia for ECT.

The present study does not support the use of TCI of propofol for anesthesia of ECT. Compared with the MI of propofol, the TCI of propofol for anesthesia of ECT had several shortcomings including higher doses of propofol, more cognitive adverse effects, longer duration of ECT procedures, and delayed recovery from anesthesia. Our preliminary results suggest that, in ECT, ‘more’ is not necessarily better than ‘less’, and sometimes ‘more’ is significantly worse than ‘less’. The use of a TCI device didn’t improve quality of care but caused potential harm to patients. Nonetheless, our study didn’t refute that TCI might be useful in special occasions. Our findings were not able to generalize to other kinds of anesthetic agents such as barbiturates and ketamine. Further research is needed to clarify the indications of the TCI for anesthesia during ECT.

### Limitations

There are several limitations in the present study. First, all subjects were psychiatric inpatients, and they were receiving different kinds of psychotropic medications. Drug interactions between propofol and psychotropic medications could be an important confounding factor. Second, the sample size was small, so the power may not be sufficient to identify differences between the two groups in terms of the efficacy and cognitive effects of ECT. Third, the present study was open-label, so bias from raters and subjects could not be eliminated. Fourth, we adopted the MoCA as the sole cognitive assessment tool in the present study. The MoCA is a measure of global cognitive function, and it may not detect subtle cognitive deficits after ECT. Finally, for individual patients, the number of ECT treatments was decided by his/her primary psychiatrist. A course of ECT treatment could have been prematurely discontinued or unduly extended. The trend analyses of outcome measures were limited by the change in sample size with the number of treatment. The number of patients who received greater than or equal 8 treatments of ECT was 16 (40%). We also examined the GEE models on this subset of the sample (supplementary Table 1). Among the patients receiving greater than or equal 8 treatments, the TCI group still had no clear advantages over the MI group. Compared with the MI group, the TCI group not only had higher total dose of propofol, but also did worse in seizure adequacy and EEG seizure duration in the later course of ECT. Therefore, our conclusion was consistent among the patients receiving greater than or equal 8 treatments.

## Conclusions

The present study does not support the use of TCI of propofol for anesthesia of ECT. Conventional manual infusion of propofol was good enough for anesthesia of ECT in terms of cost (lower dose of propofol), better indices of seizure quality, better cognitive profile, shorter duration of ECT procedures, and quicker recovery from anesthesia.

## Supplementary Information

**Additional file 1.**

## Data Availability

The datasets generated and/or analysed during the current study are not publicly available due to the restriction of IRB but are available from the corresponding author on reasonable request.

## Abbreviations

AR1: The first-order autoregressive
ASA: American Society of Anesthesiologists
ASTI: Anesthetic-ECT time interval
BMI: Body mass index
DBP: Diastolic blood pressure
ECT: Electroconvulsive therapy
EEG: Electroencephalogram
GEE: Generalized estimating equations
HR: Heart rate
MI: Manual infusion
MoCA: Montreal Cognitive Assessment
OAA/S: Observer’s Assessment of Alertness/Sedation Scale
SBP: Systolic blood pressure
TCI: Target-controlled infusion
