# Supplementary material for: Comparison of the target-controlled infusion and the manual infusion of propofol anesthesia during electroconvulsive therapy: an open-label randomized controlled trial
Source: BMC Psychiatry. 2021 Feb 4;21:71. doi: 10.1186/s12888-021-03069-6 (PMC7863537; doi:10.1186/s12888-021-03069-6)
Supplement: Supplementary file 1 — Additional file 1. [file 12888_2021_3069_MOESM1_ESM.docx]

Supplementary Table 1. The tests of model effects between groups and ECT treatment times using generalized estimating equations (GEEs) among subjects who received greater than or equal 8 treatments of ECT (N = 16).

|  | TCI versus MI | ECT treatment number | TCI × ECT treatment number versus MI × ECT treatment number | Age | Male versus Female |
| --- | --- | --- | --- | --- | --- |
|  | β (SE) | β (SE) | β (SE) | β (SE) | β (SE) |
| Total dose of propofol (mg/kg)^1^ | 2.12 (0.29)*** | 0.00 (0.01) | 0.05 (0.04) | -0.01 (0.01) | 0.16 (0.19) |
| Stimulus dose (mC) | 10.43 (18.84) | 23.95 (4.51)*** | 16.33 (5.39)** | -2.80 (1.07)** | -41.01 (32.51) |
| Recovery time (min) | 1.50 (1.96) | -0.07 (0.13) | 0.13 (0.21) | -0.02 (0.08) | 0.4­5 (2.36) |
| ASTI (min) | 0.87 (0.65) | -0.06 (0.03) | 0.03 (0.06) | 0.01 (0.02) | 0.49 (0.25) |
| Seizure adequacy (%) | 16.02 (10.88) | 1.58 (1.00) | -3.95 (1.61)* | 0.19 (0.24) | 9.31 (5.34) |
| EEG seizure duration (sec) | 18.16 (11.60) | 0.35 (0.37) | -2.74 (1.30)* | 0.33 (0.32) | 5.85 (7.60) |
| Motor seizure duration (sec) | -3.25 (6.36) | -0.59 (0.50) | 0.10 (0.80) | 0.62 (0.26)* | 8.29 (5.72) |
| Ictal HR – baseline HR _(_beats/min) | -3.23 (10.22) | 0.28 (0.81) | -0.82 (0.97) | 0.10 (0.44) | 0.37 (9.93) |
| Discharge HR - baseline HR _(_beats/min) | -8.73 (11.06) | -2.11 (0.48)*** | 0.05 (0.96) | -0.27 (0.29) | -3.10 (7.94) |
| Ictal SBP– baseline SBP (mmHg) | 9.07 (9.74) | 2.65 (0.86)** | -0.27 (1.15) | -0.43 (0.18)* | -23.18 (4.92)*** |
| Discharge SBP– baseline SBP (mmHg) | 12.35 (10.81) | 1.18 (0.45)** | -0.69 (0.98) | -0.47 (0.26) | -24.72 (6.99)*** |
| Ictal DBP– baseline DBP (mmHg) | -1.56 (5.54) | -055 (0.57) | 1.53 (0.70)* | -0.33 (0.19) | -11.24 (4.02)** |
| Discharge DBP– baseline DBP (mmHg) | 5.06 (6.18) | -0.07 (0.32) | -0.65 (0.52) | -0.49 (0.16)* * | -10.94 (3.86)** |

*: p < 0.05; **: p < 0.01; ***: p < 0.001

^1^: Total dose of propofol was total amount of propofol divided by ideal body weight.

ASTI: anesthetic-stimulation time interval; EEG: electroencephalogram; HR: heart rate; SBP: systolic blood pressure; DBP: diastolic blood pressure.
